# Distinct patterns of neural response to faces from different races in humans and deep networks

**DOI:** 10.1093/scan/nsad059

**Published:** 2023-10-14

**Authors:** Ao Wang, Magdalena W Sliwinska, David M Watson, Sam Smith, Timothy J Andrews

**Affiliations:** Department of Psychology, University of York, York YO10 5DD, UK; Department of Psychology, University of Southampton, Southampton SO17 1BJ, UK; Department of Psychology, University of York, York YO10 5DD, UK; School of Psychology, Liverpool John Moores University, Liverpool L2 2QP, UK; Department of Psychology, University of York, York YO10 5DD, UK; Department of Psychology, University of York, York YO10 5DD, UK; Department of Psychology, University of York, York YO10 5DD, UK

**Keywords:** face, race, DCNN, ORE

## Abstract

Social categories such as the race or ethnicity of an individual are typically conveyed by the visual appearance of the face. The aim of this study was to explore how these differences in facial appearance are represented in human and artificial neural networks. First, we compared the similarity of faces from different races using a neural network trained to discriminate identity. We found that the differences between races were most evident in the fully connected layers of the network. Although these layers were also able to predict behavioural judgements of face identity from human participants, performance was biased toward White faces. Next, we measured the neural response in face-selective regions of the human brain to faces from different races in Asian and White participants. We found distinct patterns of response to faces from different races in face-selective regions. We also found that the spatial pattern of response was more consistent across participants for own-race compared to other-race faces. Together, these findings show that faces from different races elicit different patterns of response in human and artificial neural networks. These differences may underlie the ability to make categorical judgements and explain the behavioural advantage for the recognition of own-race faces.

## Introduction

The ability to categorize people based on the appearance of the face plays an important role in our daily social interactions. These decisions can often lead to stereotypical judgements about a person or be used as a basis for group membership (Tajfel *et al.*, 1971; Macrae and Bodenhausen, 2000). A range of evidence shows that we are easily and automatically able to perceive the race of faces (Ellis *et al.*, 1975; Shepherd and Deregowski, 1981; Hill *et al.*, 1995; Yan *et al.*, 2017). Our ability to accurately discriminate faces according to race shows that they have statistically different visual properties (Farkas *et al.*, 2005). The physical differences associated with race are evident in the average shape, as well as in hair and skin colour (Farkas *et al.*, 2005). Behavioural studies have shown that both shape and colour are used in perceptual judgements of race (Hill *et al.*, 1995). However, it is less clear how these differences in facial appearance are represented in human and artificial neural networks.

Differences in race can influence our ability to recognize faces—a phenomenon known as the other race effect—ORE (Malpass and Kravitz, 1969; Meissner and Brigham, 2001). Neuroimaging studies that have investigated the effect of face race have focussed on the ORE (Natu and O’Toole, 2013; Molenberghs and Louis, 2018; Bagnis *et al.*, 2020). Some studies have found a larger fMRI response in face-selective regions to own-race faces (Golby *et al.*, 2001; Feng *et al.*, 2011; Natu *et al.*, 2011), others report that a larger response to own-race faces is dependent on the task or type of image (Cunningham *et al.*, 2004; Lieberman *et al.*, 2005; Kim *et al.*, 2006), whereas others find no difference in response to own-race and other-race faces (Hart *et al.*, 2000; Brosch *et al.*, 2013; Ratner *et al.*, 2013). Other studies have used fMR adaptation—the reduced response to repeated exposures of the same stimulus (Grill-Spector *et al*., 1999; Andrews and Ewbank, 2004; Ewbank and Andrews, 2008; Andrews *et al.*, 2010). These studies have reported greater adaptation to own-race faces compared to other-race faces in the fusiform face area (FFA) (Hughes *et al.*, 2019; Reggev *et al.*, 2020) that suggest differences in the time-scale of response to own-race and other-race faces (Natu *et al.*, 2011).

Fewer studies have directly explored the question of whether there are different patterns of responses to faces from different races. Multi-voxel pattern analysis has shown differences in the pattern of response to Asian and White faces across regions of the temporal lobe, including the fusiform gyrus (Natu *et al.*, 2011). Distinct spatial patterns of response in face responsive regions of the occipital and temporal lobes have also been reported for Black and White faces (Ratner *et al.*, 2013), although this is most evident in participants with significant own-race bias (Brosch *et al.*, 2013).

Differences in processing faces from different races have been shown by algorithms trained to recognize faces. For example, several studies have found that many algorithms have different levels of recognition for faces from different races (Furl *et al.*, 2002; Phillips *et al.*, 2011; Natu and O’Toole, 2013). This is typically explained by a bias toward White or Caucasian faces, reflecting the images that are used to train the algorithm. In recent years, deep convolutional neural networks (DCNN) have surpassed previous face recognition algorithms in their ability to make accurate judgements across a range of natural viewing conditions (Parkhi *et al.*, 2015; O’Toole *et al.*, 2018). DCNNs also show a bias toward White faces, which again reflects the bias in the images used during training (Cavazos et al., 2020). Nevertheless, this bias toward White faces can be reversed if the DCNN is trained on non-White faces (Tian *et al.*, 2021).

The aim of this study was to explore the ability to discriminate race in deep neural networks and face-selective regions of the human brain. Previous studies have typically explored this question using either behavioural, neural or computational methods using different image sets. Differences in the interpretation could therefore reflect differences in methodological approach or images used. In this study, we used neuroimaging, computational and behavioural methods with the same image set of Asian, Black and White faces to ask a number of intersecting questions that explore the way that face race is represented in humans and artificial neural networks.

In the first analysis, we asked whether there were distinct patterns of response to faces from different races in a DCNN trained to discriminate faces. Given the differences in image properties evident in faces from different races, the expectation was that these differences would be evident in the DCNN. However, it is not clear whether these differences would be most evident in the earlier convolutional layers that reflect the low-level image properties or in the later fully connected layers at which the representation of identity emerges. Next, we asked whether the behavioural biases in humans to categorize or individuate own-race and other-races faces are reflected in the output of the DCNN. Given the bias toward white faces during training, we predicted a corresponding bias in the output of the DCNN.

In the second analysis, we investigated neural patterns of response to faces from different races in face-selective regions. We recruited a large sample of Asian and White participants and measured neural responses to faces from the same image set using fMRI. We asked if there were distinct patterns of neural response in face-selective regions to faces from different races. We also asked if the patterns of response were more distinct for own-race faces compared to other-race faces. Finally, we used fMR adaptation to determine if there was an own-race bias in the individuation of faces. In a control analysis, we compared patterns of response to pareidolic objects that give rise to the perception of a faces. Our prediction was that there should be no effect of participant race, because both sets of participants would have had similar exposure to inanimate objects.

## Methods

### Stimuli

**Fig. 1.**
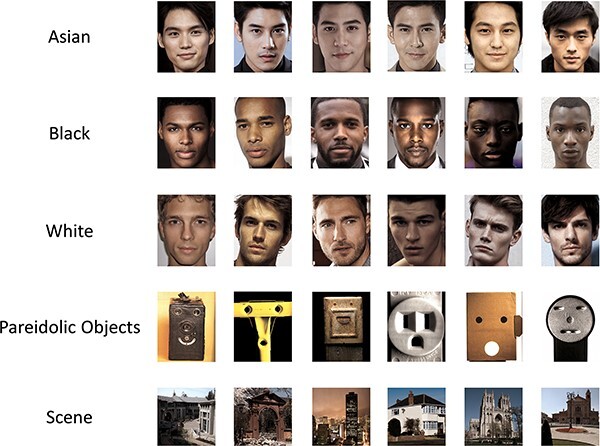
Examples of images from the different stimulus conditions.

Examples of the images are shown in Figure 1. Face images were taken from a behavioural study that showed the ORE in a large group of Asian and White participants (Wang *et al.*, 2022). In this study, there were three face matching tasks using either Asian, Black or White male faces. Each matching task had 90 trials. In each trial, a pair of face images was presented together. In half of the trials, the faces were from the same identity and in the remaining half of the trials the faces were from two different identities. Pareidolic objects were also taken from a range of freely available internet sources. Scene images were drawn from indoor, outdoor man-made natural stimuli from the Scene Understanding (SUN) database (Xiao *et al.*, 2010).

### Deep convolutional neural network

We used the VGG-Face DCNN trained to discriminate facial idenity (Parkhi *et al.*, 2015). We compared each pair of face images from all three matching tasks. We used the automatic face detection algorithm packaged with VGG-Face to crop images to a square bounding box around the face, after which images were resized to 224 × 224 for input into the DCNN. The DCNN consists of 13 convolutional layers and 3 fully connected (Fc) layers, which were used for the analysis. Each convolutional layer is followed by one or more non-linear layers, such as rectified linear units or max pooling, which were not used in this analysis. The dimensions of the layers are as follows: Conv1 = Conv1 = 224 × 224 × 64 = 3 211 264; Conv2 = 112 × 112 × 128 = 1 605 632; Conv3 = 56 × 56 × 256 = 802 816; Conv4 = 28 × 28 × 512 = 401 408; Conv5 = 14 × 14 × 512 = 100 352; Fc6 = 4096; Fc7 = 4096; Fc8 = 2622. The DCNN was trained on over 2.6 M face images from over 2.6 K identities. Face recognition on the Labeled Faces in Wild dataset (Huang *et al*., 2008) and YouTube Faces (Wolf *et al.*, 2011) for VGG-Face is 99.9% and 97.4%, respectively.

To determine which layers of the DCNN show a higher similarity for faces of the same race compared to faces of different races, we measured the similarity between the feature vectors of all pairs of face images within each DCNN layer. To do this, the activations for each face from a given layer were flattened into vectors and then correlated. To determine which layers best predict behaviour, we measured the representational similarity (Kriegeskorte, 2008) for all pairs of faces on the matching task using the DCNN. Behavioural measurements were taken from a previous study in which Asian and White participants were asked to indicate whether pairs of Asian, Black or White faces were from the same identity or a different identity (Wang *et al.*, 2022). Behavioural similarity matrices were constructed by calculating the proportion of same responses for each of the 90 trials in each task across Asian or White participants. The behavioural similarity values were then correlated against the feature correlations for the corresponding 90 face pairs within each of the DCNN similarity matrices.

We tested the ability of the DCNN representations to decode both the race and identity of the faces. In each case, we employed two approaches: one based on signal detection theory, and another based on parametric tests of the correlations themselves. We first decoded the race of the faces. Using a signal detection theory approach, we used a one-versus-rest strategy where we tested the ability to decode each of the three target races against the remaining two races combined. For each of the 540 faces, we calculated the average correlation to other faces from the target race (excluding any comparisons between an image and itself). We then defined all faces from the target race as belonging to the positive class and all faces from the other races as belonging to the negative class. We would predict higher correlations within the positive than negative class if the target race can be decoded successfully. We measured decoding sensitivity by calculating the area under the receiver operating characteristic (ROC) curve. We converted this to area under the curve (AUC) to a value of d’ according to the formula $d{^{^{\prime}}} = \,\sqrt 2 \times {{\Phi}^{ - 1}}\left( {{\mathrm{AUC}}} \right)$, where ${{\Phi}^{ - 1}}$ is the inverse of the standard normal cumulative distribution function. This process was then repeated for each layer of the DCNN, and then again selecting each race as the target race in turn. To compare decoding sensitivity against chance, we performed a maximum statistic permutation test. For a given target race, on each permutation, the order of the class labels was permuted and the d’ scores recalculated for each layer of the DCNN, and the maximum score over all layers was recorded. This was repeated for 10 000 permutations to build an empirical null distribution that controls for the familywise error rate over DCNN layers. These permutations were then repeated for each race in turn. One-tailed *P*-values were estimated by the proportion of scores in the null distributions falling above the true d’ prime values—these *P*-values were then further Bonferroni corrected for the three races. Second, we performed parametric analyses of the correlation values themselves. For each face, we calculated the average correlation to other faces from the same race and to other faces from the other races, then took the difference between these values—this yielded an average ‘within > between race’ value for each image. These difference values were then entered into a two-way mixed-design ANOVA with a repeated-measures factor the DCNN layer (16 levels) and an independent-samples factor for the face race (Asian, Black, White). A Greenhouse–Geisser sphericity correction was applied to all effects. We also compared the correlations for each layer and race separately via a series of one-tailed paired-samples *t*-tests contrasting within-race greater than between-race; a Bonferroni–Holm correction for multiple comparisons was applied over the 48 layer and race combinations.

Next, we tested the ability to decode face identity. We selected the correlations for the 270 image pairs presented in the behavioural experiment (45 same-identity and 45 different-identity pairs per each of the three races). We would predict higher correlations for the same- than different-identity pairs if the identity can be decoded successfully. We first employed the signal detection theory approach. For a given race, we calculated the area under the ROC curve for decoding same versus different identity pairs based on their correlations, which was then converted to a d’ value. This was repeated for each race and DCNN layer in turn. We again employed a maximum statistic permutation test to compare decoding sensitivity against chance. For each race, the same/different-identity class labels were permuted 10 000 times. Familywise error corrected one-tailed *P*-values were derived from the empirical null distributions, which were then further Bonferroni corrected over the three races. Finally, we employed parametric tests of the correlations themselves. The correlations were entered into a three-way mixed-design analysis of variance (ANOVA) with a repeated-measures factor for DCNN layer (16 levels) and between-subjects factors for the identity-pairing (same, different) and race (Asian, Black, White). We additionally compared the correlations for each layer and race separately via a series of one-tailed independent-samples *t*-tests contrasting same-identity greater than different-identity; a Bonferroni–Holm correction was applied over the 48 layer and race combinations.

### fMRI experiment

A sample of 28 East Asians (19 females, mean age = 22.0, SD = 3.0 years) and 29 Whites (20 females, mean age = 21.6, SD = 3.4 years) participants were recruited for this study from the staff and student population at the University of York. East Asian and White participants had grown up in East Asian or Western European countries, respectively. For Asian participants, the average stay-in UK period was less than a year (mean ±SEM: 10.7 ±0.57 months). All participants gave their written informed consent. All participants had normal or corrected to normal vision. The study was approved by the York Neuroimaging Centre (YNiC) Ethics Committee.

Neural responses were measured using fMRI, while participants viewed images from four conditions (Asian face, Black face, White face, Pareidolic object). Images from these conditions were presented in a blocked design in two arrangements (Same Image, Different Image). Each block was 6 s in duration and was composed of 6 images. Each image was presented for 800 ms presentation with a 200 ms inter-stimulus-interval. Blocks were separated with 9 s fixation screen. In Same Image blocks, a single image of the same face was presented six times, whereas in Different Image blocks, six different identity images were presented. The order of blocks and images were pseudo-randomized. Each stimulus condition was repeated five times. We also included a control condition (Scene) to define the face regions.

Images were superimposed on a mid-gray background and had a visual angle of ∼10.7°. They were back-projected onto a custom in-bore acrylic screen at a distance of 57 cm from the participant. Stimulus presentation was controlled through Psychopy (Peirce *et al.*, 2019). To avoid any confounds with task difficulty, participants performed an orthogonal non-face task in which they pressed a button with their right index finger on a response box whenever a green fixation cross appeared. Green fixation crosses occurred at random times during the stimulus presentation.

The structural and functional data were collected at the York Neuroimaging Centre with a 3T Siemens Magnetom Prisma MRI scanner (Siemens Healthcare, Erlangen, Germany) and a 20-channel-phased array head coil. A gradient-echo echo-planar imaging (EPI) sequence was used to collect the functional data from 60 contiguous axial slices [repetition time (TR) = 3000 ms, echo time (TE) = 35 ms, FoV = 240 × 240 mm, matrix size = 80 × 80, voxel size = 3 × 3 × 3 mm, flip angle = 90] that provided whole-brain coverage. T-1-weighted MPRAGE anatomical scans were also acquired for anatomically localizing functional activation. The structural data were recorded via matrix of 176 × 256 × 256 and voxel size 1 × 1 × 1 mm, with repetition time (TR) = 2300 ms, and echo time (TE) = 2.26 ms.

The fMRI data were analysed using the fMRI Expert Analysis Tool (FEAT) v6.0 (http://www.fmrib.ox.ac.uk/fsl). Motion correction was achieved via MCFLIRT, FSL (http://www.fmrib.ox.ac.uk/fsl). Slice-timing correction also applied and followed by temporal high-pass filtering (Gaussian-weighted least squares straight line fittings, sigma = 50 s). Spatial smoothing (Gaussian, FWHM 5 mm) and pre-whitening were applied to remove temporal auto-correction. For each condition, we generated parameter estimates by regressing the hemodynamic response of each voxel against a box-car that was convolved with a single-gamma haemodynamic response function. Functional data were registered to a high-resolution T1-anatomical image, and then onto the standard Montreal Neurological Institute (MNI) brain (ICBM152).

**Fig. 2.**
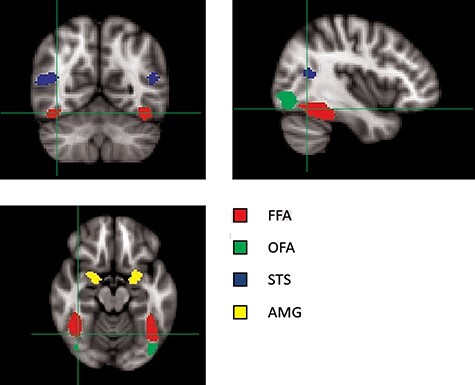
Location of the face-selective regions following a group analysis across all participants. Regions of interest are superimposed on the MNI152 brain (*x* = 40, *y* = -−60, *z* = −16). FFA: fusiform face area, OFA: occipital face area, STS: posterior superior temporal sulcus, AMG: amygdala.

We defined regions of interest (ROIs) across the brain using fMRI data from both Asian and White participants (see also Golby *et al.*, 2001; Feng *et al.*, 2011; Hughes *et al.*, 2019; Reggev *et al.*, 2020). To define the ROIs, the response to all face conditions (Asian, Black and White) was contrasted with the response to scenes (Figure 2). This allowed the definition of the face-selective regions: fusiform face area (FFA), occipital face area (OFA), superior temporal sulcus (STS) and amygdala (AMG). The peak face-selective and scene-selective voxels (i.e. those with the highest *z*-value) were identified and a flood fill algorithm was used to identify a cluster of 500 spatially contiguous voxels for each ROI to a lower threshold of *z* > 2.3 (Weibert and Andrews, 2015). If it was not possible to define a 500 voxel ROI for a region, the region was defined by the largest size to the nearest 100 (Table 1). The 500 voxel ROIs were found bilaterally for the FFA and OFA. It was possible to define a 500 voxel ROI in the right STS, but only 200 voxels ROI in the left STS. The AMG was defined by 200 voxel ROIs in the left and right hemisphere.

**Table 1.** MNI coordinates (mm) of peak voxels in face-selective regions

ROI	Hemisphere	*x*	*y*	*z*	Voxels	Face > scene (*z*)
FFA	Left	−42	−62	−16	500	6.4
	Right	44	−52	−18	500	7.9
OFA	Left	−40	−80	−10	500	6.3
	Right	48	−76	2	500	8.1
STS	Left	−48	−64	12	200	3.2
	Right	48	−76	8	500	6.1
AMG	Left	−20	− 4	−14	200	3.5
	Right	20	− 4	−10	200	4.7

For the multi-voxel pattern analysis (MVPA), parameter estimates for each different identity condition were normalized by subtracting the mean response across all different identity conditions for each voxel. For each pairwise combination of conditions, the pattern of response in each participant was compared with the corresponding group pattern with the remaining participants in their group (Asian or White). This leave-one-participant-out (LOPO) cross-validation paradigm was repeated for each participant for each combination of conditions (Rice *et al.*, 2014). The MVPA was implemented using the PyMVPA toolbox (http://www.pymvpa.org; Hanke *et al.*, 2009). The Pearson correlation coefficients were then used to calculate the representational similarity in the patterns of response to different conditions. A Fisher’s *z*-transformation was then applied to the correlations prior to further statistical analysis.

For the adaptation analysis, we compared the peak responses for the Same-Identity and Different-Identity conditions in each ROI. To determine whether the magnitude of adaptation varied across different race faces and for different race participants, a mixed-design ANOVA was performed on each of the core face-selective regions comprising a between-subjects factor of participant race (Asian, White), and repeated-measures factors of Face Race (Asian, Black, White) and Adaptation (Same, Different).

## Results

### DCNN analysis

**Fig. 3.**
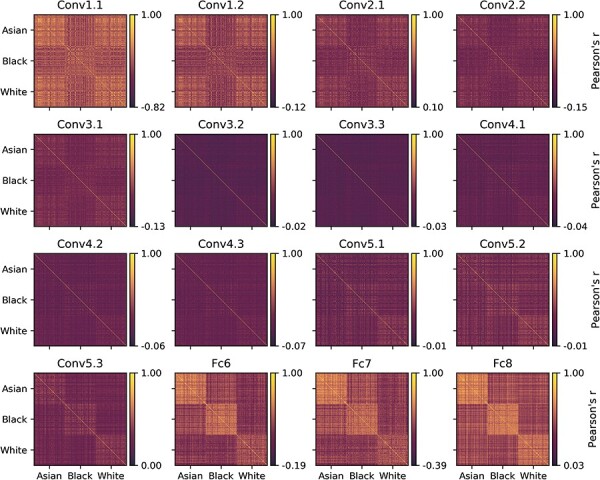
Similarity matrices from the images in the face matching task calculated from the 13 convolutional and 3 fully connected layers of VGG-Face. The similarity matrix shows the similarity (correlation) of all combinations of the 540 images in the stimulus set. The 540 images comprised 180 Asian, 180 Black and 180 White faces. The similarity of each image pair was calculated by correlating the DCNN feature vectors for pairs of images.

We used a pre-trained DCNN (VGG-Face) to compare faces from different races. Figure 3 shows the similarity matrices from the convolutional and fully connected layers across all 540 face images. This shows that the differences in similarity between faces from different races become most evident in the fully connected layers. In the fully connected layers, the within-race versus between-race difference was similar for Asian, Black and White faces. To determine whether faces from the same race (within) were more similar than faces from different races (between), we ran a *t*-test for each face race. For example, to show the categorization effect for Asian faces, the correlations for all combinations of Asian faces were compared to all combinations of Asian and Black or Asian and White faces. The statistical differences are shown for each layer in Table 2. An ANOVA on the within–between values revealed not only a main effect of Race [*F*(2537) = 5.49, *P* = 0.004] and Layer [*F*(2.21,1189.4) = 1530.4, *P* < 0.001], but also a Race*Layer interaction [*F*(4.42,1189.4) = 105.2, *P < *0.001].

**Table 2.** Same-race/different-race comparison from the output of VGG-Face

	Asian		Black		White	
Layers	*t*	*P*	*t*	*P*	*t*	*P*
Conv1.1	53.73	0.001	31.03	0.001	34.26	0.001
Conv1.2	53.76	0.001	21.84	0.001	37.50	0.001
Conv2.1	43.28	0.001	27.13	0.001	35.00	0.001
Conv2.2	40.30	0.001	32.41	0.001	34.17	0.001
Conv3.1	36.54	0.001	32.01	0.001	43.16	0.001
Conv3.2	11.45	0.001	19.74	0.001	50.61	0.001
Conv3.3	1.03	0.301	25.28	0.001	49.80	0.001
Conv4.1	−3.82	0.001	26.48	0.001	50.98	0.001
Conv4.2	0.32	0.749	24.75	0.001	45.03	0.001
Conv4.3	2.16	0.031	27.76	0.001	41.15	0.001
Conv5.1	1.02	0.307	27.57	0.001	53.40	0.001
Conv5.2	11.89	0.001	37.77	0.001	61.18	0.001
Conv5.3	63.10	0.001	74.49	0.001	105.85	0.001
Fc6	185.18	0.001	177.87	0.001	120.28	0.001
Fc7	200.93	0.001	129.33	0.001	94.37	0.001
Fc8	171.61	0.001	177.66	0.001	158.92	0.001

**Fig. 4.**
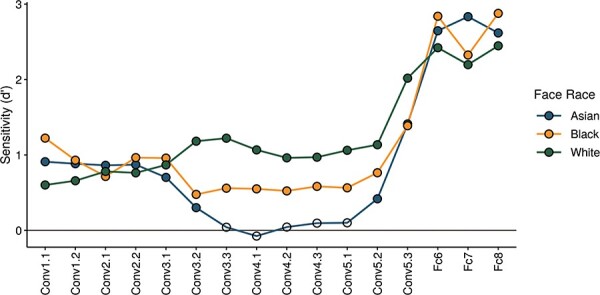
Sensitivity to decoding face races across different layers of VGG-Face. Filled symbols indicate decoding significantly higher than chance. Sensitivity to face race becomes most evident in the fully connected layers (Fc). However, there is also a greater sensitivity to White faces compared to Black and Asian faces in the later convolutional layers (Conv) of the DCNN.

Next, we compared the categorization effect for faces from different races across different layers of the DCNN. Figure 4 (and Supplementary Figure S1) shows the sensitivity (d’) for decoding each face-race against the other races across all layers of the DCNN. This shows that highest sensitivity to race was evident in the fully connected layers of the DCNN. However, we also found that the greatest differences in sensitivity to different race faces were evident in the later convolutional layers. Interestingly, the DCNN is more sensitive to White faces compared to Asian and Black faces in these convolutional layers of the DCNN (Conv3.2—Conv5.3).

A well-established behavioural effect is that human participants are able to individuate own-race faces more efficiently than other-race faces (Malpass and Kravitz, 1969; Meissner and Brigham, 2001). Accordingly, we asked whether there were differences in the ability of the DCNN to discriminate identity across the different races. We focused on the 90 face pairs used in each matching task (see Wang *et al.*, 2022). For each task, there were 45 same identity trials and 45 different identity trials. In each layer of the DCNN, we correlated the feature vectors between the identity pairs for each trial, then compared the similarity of the faces from the same identity trials against those from the different identity trials. Higher correlations were observed for same-identity compared to different-identity pairings in the later DCNN layers (peaking in fully connected layer 7), reflecting decoding of facial identity. We entered the correlations into a three-way mixed-design ANOVA with independent-samples factors for the identity-pairing (same, different) and race (Asian, Black, White) and a repeated-measures factor for the DCNN-layer (1–16). This revealed a significant main effect of DCNN-layer [*F*(2.79, 736.30) = 872.11, *P* < 0.001, $\eta _P^2$ = 0.77, $\eta _G^2$ = 0.62], but no significant main effects of identity-pairing [*F*(1, 264) = 1.31, *P* = 0.254, $\eta _P^2$ < 0.01, $\eta _G^2$ < 0.01] or race [*F*(2, 264) = 0.12, *P* = 890, $\eta _P^2$ < 0.01, $\eta _G^2$ < 0.01]. Importantly, there were significant identity-pairing * DCNN-layer [*F*(2.79, 736.30) = 49.47, *P* < 0.001, $\eta _P^2$ = 0.16, $\eta _G^2$ = 0.09] and identity-pairing * race [*F*(2, 264) = 6.72, *P* = 0.001, $\eta _P^2$ = 0.05, $\eta _G^2$ = 0.03] interactions. There was also a significant race*DCNN-layer interaction [*F*(5.58, 736.30) = 3.80, *P* = 0.001, $\eta _P^2$ = 0.03, $\eta _G^2$ = 0.01]. Finally, the three-way identity-pairing by race by DCNN-layer approached significance [*F*(5.58, 736.30) = 1.87, *P* = 0.088, $\eta _P^2$ = 0.01, $\eta _G^2$ < 0.01].

**Fig. 5.**
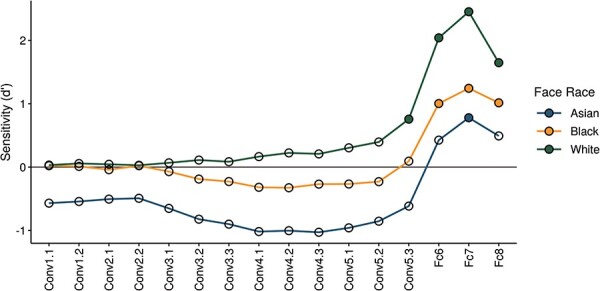
Decoding of facial identity in VGG-Face. Plot illustrates the sensitivity to decoding same-identity versus different-identity face pairs for each layer in each of the different races. Filled symbols indicate significantly higher sensitivity to same-identity than different-identity face pairs. The ability to discriminate identity was greatest in the fully connected layers (Fc) and there was a greater sensitivity to White faces compared to Asian and Black faces.

To further investigate the decoding of facial identity, we calculated the ability of the DCNN to discriminate same-identity and different-identity faces. Figure 5 (and Supplementary.Figure S2) shows the sensitivity (d’) for decoding each face race against the other races across all layers of the DCNN. This shows that highest sensitivity to identity was evident in the fully connected layers of the DCNN. We also found that the sensitivity to identity was greatest for White faces in the fully connected layers. In summary, facial identity was decoded best in the fully connected DCNN layers. However, decoding accuracy was best for White faces, next best for Black faces and worst for Asian faces.

**Fig. 6.**
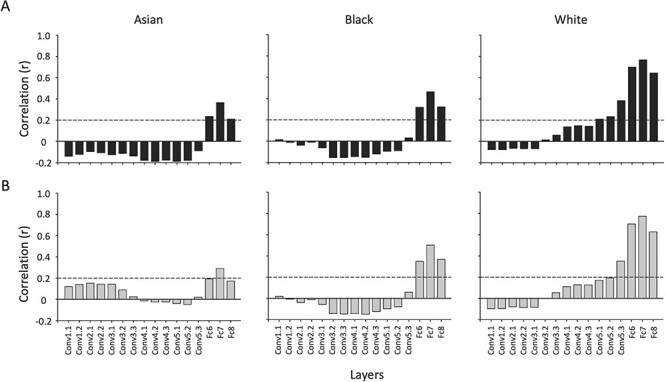
(A) The correlation between pairwise image similarity in the DCNN and proportion of same identity judgements of Asian participants and (B) White participants was calculated for different layers in the DCNN. The dashed line indicates the critical *r*-value at *P* < 0.05. Significant correlations were most evident in the fully connected layers (14–16).

We then asked whether human performance on the matching tasks correlated with the representations within each layer of the DCNN. Again, this analysis focused on the 90 trials in each task (see Wang *et al.*, 2022). In this study, 70 Asian participants and 70 White participants made same identity or different identity judgements on the pairs of Asian, Black and White faces. Similarity between each face pair in each layer of the DCNN was correlated with proportion of same identity judgements for Asian and White participants (Figure 6). We found that similarity in early convolutional layers of the DCNN did not predict behaviour. However, we found significant correlations in the fully connected layers for all three races. Interestingly, the correlation between behaviour and DCNN similarity was greatest for White faces. The highest correlations were evident in Fc7 (layer 15 in the DCNN). A Fisher’s *z* comparison of the correlations shows that were significantly higher correlations in Fc7 between White faces and Asian faces (Asian participants: *z* = 4.16, *P* < 0.0001; White participants: *z* = 4.81, *P* < 0.0001) and between White faces and Black faces (Asian participants: *z* = 3.37, *P* < 0.001; White participants: *z* = 3.15, *P* = 0.002). However, there was no difference between Asian and Black faces (Asian participants: *z* = 0.79, *P* = 0.429; White participants: *z* = 1.66, *P* = 0.09).

To summarize the DCNN analysis, we show the following key findings: (I) the categorization of face race and the ability to discriminate identity is greater in the fully connected layers; (II) the categorization of White faces is more efficient than Asian and Black faces in the later convolutional layers; (III) the identification of White faces is more efficient than Asian and Black faces; (IV) identity judgements from human participants are more correlated with the output of the fully connected layers with White compared to Asian and Black faces.

### fMRI analysis

**Fig. 7.**
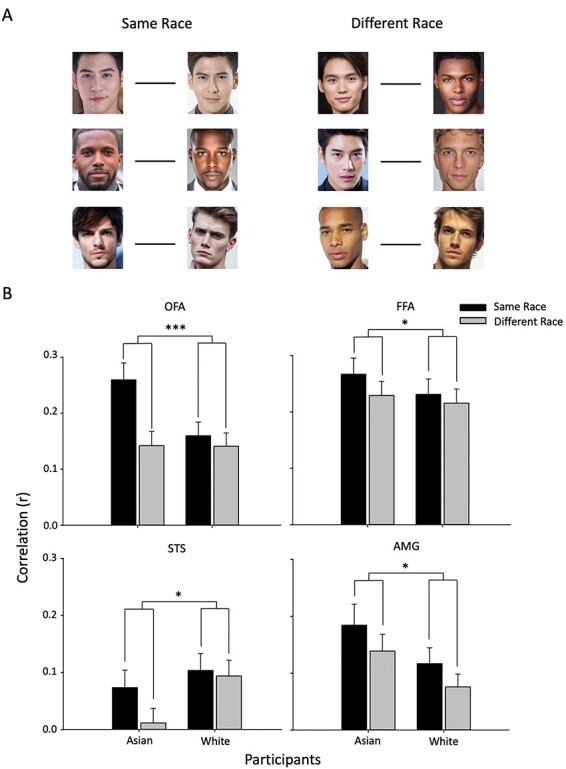
MVPA showing different spatial patterns of response to faces from different races in face regions. (A) The similarity in the spatial patterns of response between faces from the Same Race was compared to the similarity in the spatial patterns between faces from Different Races. (B) This shows a main effect of Face Race in all regions with more similar patterns of response to faces from the Same Race compared to Different Race (*** *P* < 0.001, * *P* < 0.05). Error bars represent standard error of the mean.

First, we asked if there were distinct spatial patterns of response to faces from different races, irrespective of whether they are own-race or other-race. Figure 7 shows the similarity in the patterns of response to faces from the same race (Asian–Asian, Black–Black, White–White) compared with the similarity in the patterns of response to faces from different races (Asian–Black, Asian–White, Black–White). A Face Race (Same Race, Different Race) * Participant Race (Asian, White) repeated measure ANOVA was then performed for each ROI.

There was a significant main effect of Face Race in the OFA [*F*(1, 55) = 31.097, *P* < 0.001, η_G_^2^ = 0.361] as well as a significant interaction between Face Race and Participant Race [*F*(1, 55) = 16.378, *P* <0.001, η_G_^2^ = 0.229). Planned comparisons showed a significant difference between same-race and different-race faces in both Asian (*t* = 5.311, *P* < 0.001, Cohen’s *d* = 0.795) and White (*t* = 1.708, *P* < 0.05, Cohen’s *d* = 0.267) participants. In the FFA, there was a significant main effect of Face Race [*F*(1, 55) = 6.245, *P* = 0.015, η_G_^2^ = 0.102], but no interaction between Face Race*Participant Race [*F*(1, 55) = 1.111, *P* = 0.296, η_G_^2^ = 0.020]. Planned comparisons showed a significant difference between same-race and different race in Asian participants (*t* = 2.063, *P* = 0.049, Cohen’s *d* = 0.415) and a marginal effect for White participants (*t* = 1.380, *P* = 0.090, Cohen’s *d* = 0.259). In the STS, there was a significant main effect of Face Race [*F*(1, 55) = 5.335, *P* = 0.025, η_G_^2^ = 0.088], while the Face Race*Participant Race interaction was not significant [*F*(1, 55) = 2.843, *P* = 0.097, η_G_^2^ = 0.049]. Planned comparisons showed a significant difference between same-race and different race in Asian participants (*t* = 2.562, *P* = 0.008, Cohen’s *d* = 0.145), but no difference for White participants (*t* = 0.494, *P* = 0.313, Cohen’s *d* = 0.109). In the AMG, there was a significant main effect of Face Race [*F*(1, 55) = 4.799, *P* = 0.033, η_G_^2^ = 0.080], but no Face Race*Participant Race interaction [*F*(1, 55) = 0.013, *P* = 0.909, η_G_^2^ < 0.0001]. Planned comparisons showed a marginal effect between same-race and different-race faces in the Asian participants (*t* = 1.604, *P* = 0.06, Cohen’s *d* = 0.062) and a marginal effect in White participants (*t* = 1.492, *P* = 0.073, Cohen’s *d* = 0.300).

**Fig. 8.**
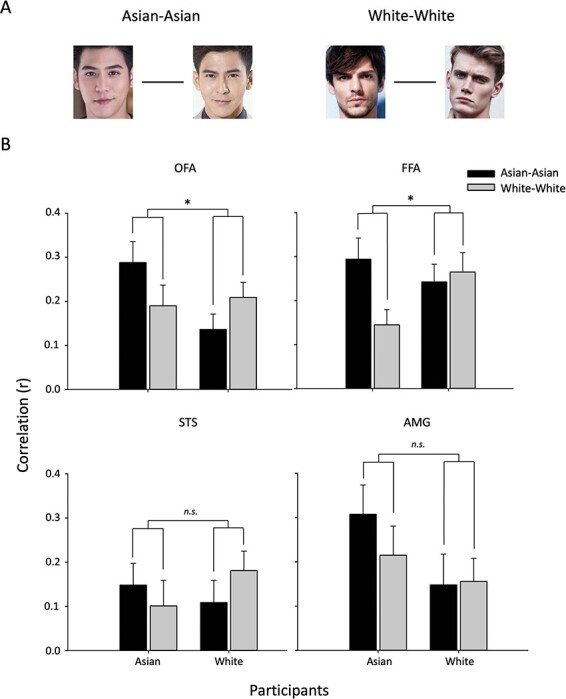
MVPA showing more similar spatial patterns of response to own-race compared to other-race faces in the OFA and FFA. (A) The spatial pattern of response between White or between Asian faces was compared in Asian and White participants. (B) There was an interaction between Face and Participant race in the OFA and FFA (*P < 0.05, n.s. not significant). This reflects the spatial pattern of response to own-race faces being more similar than the pattern of response to other-race faces in these regions. Error bars represent standard error of the mean.

We then asked whether the spatial patterns of response were more distinct for own-race faces compared to other-race faces (Figure 8). To address this question directly, we restricted the analysis to Asian and White faces and performed a Face (Asian–Asian, White–White) * Participant Race (Asian, White) ANOVA. In the OFA, there was an interaction between Face*Participant [*F*(1, 55) = 5.234, *P* = 0.026, η_G_^2^ = 0.087]. This reflected a larger effect for White faces in White participants [*t*(28) = 1.708, *P* = 0.049, Cohen’s *d* = 0.682] and a marginal effect for Asian faces in Asian participants [*t*(27) = 1.585, *P* = 0.062, Cohen’s *d* = 0.385]. In the FFA, there was an interaction between Face*Participant [*F*(1, 55) = 4.261, *P* = 0.044, η_G_^2^ = 0.072]. This reflected a larger effect for Asian faces in Asian participants [*t*(27) = 2.816, *P* = 0.004, Cohen’s *d* = 0.168], but no corresponding larger effect for White faces in White participants [*t*(28) = 0.349, *P* = 0.365, Cohen’s *d* = 0.265]. There was no significant difference between Face * Participant in the STS [*F*(1, 55) = 1.939, *P* = 0.169, η_G_^2^ = 0.034] or the AMG [*F*(1, 55) = 0.544, *P* = 0.460, η_G_^2^ = 0.010]. This shows that there was a significant difference in the pattern of response between own-race and other-race faces in the OFA and FFA, but not in the STS and AMG.

**Fig. 9.**
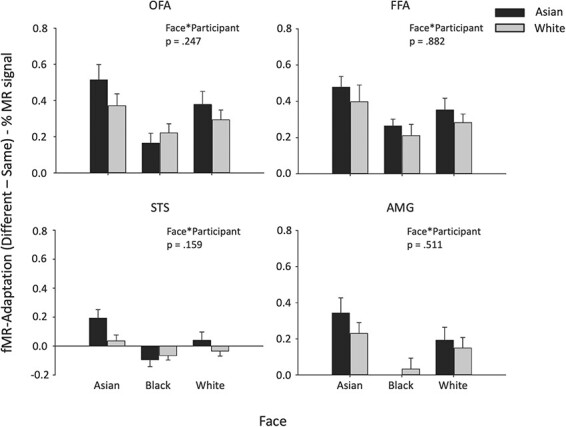
fMR adaptation to faces from different races. There were no significant interactions between Face*Participant in any of the face-selective regions. This shows that the magnitude of adaptation was not modified by the race of the participants. Error bars represent SEM.

Next, we asked whether adaptation was greater for own-race faces compared to other-race faces in the face-selective regions of human participants (Figure 9). A mixed-design ANOVA was performed on each of the core face-selective regions comprising a between-subjects factor of Participant Race (Asian, White), and repeated-measures factors of Face Race (Asian, Black, White) and Adaptation (Same, Different). There was a significant main effect of Adaptation in the OFA [*F*(1, 55) = 127.57, *P* < 0.001, η_G_^2^ = 0.699], FFA [*F*(1, 55) = 131.61, *P* < 0.001, η_G_^2^ = 0.705] and AMG [*F*(1, 55) = 28.28, *P* < 0.001, η_G_^2^ = 0.336], but not in the STS [*F*(1, 55) = 0.444, *P* = 0.508, η_G_^2^ = 0.008]. There was also a significant Face*Adaptation interaction in each region [FFA: *F*(2, 110) = 5.906, *P* = 0.004, η_G_^2^ = 0.097; OFA: *F*(2, 110) = 8.477, *P* < 0.001, η_G_^2^ = 0.134; STS: *F*(2, 110) = 8.595, *P* < 0.001, η_G_^2^ = 0.135; AMG: *F*(2, 110) = 8.635, *P* < 0.001, η_G_^2^ = 0.136]. This shows that adaptation varied according to the stimulus set, with higher adaptation to Asian faces. However, there was no Face*Participant interaction for Adaptation in any of the face regions [FFA: *F*(2, 110) = 0.125, *P* = 0.882, η_G_^2^ = 0.002], OFA: *F*(2, 110) = 1.418, *P* = 0.247, η_G_^2^ = 0.025, STS: *F*(2, 110) = 1.868, *P* = 0.159, η_G_^2^ = 0.033, AMG: *F*(2, 110) = 0.676, *P* = 0.511, η_G_^2^ = 0.012]. Together, this shows that adaptation to different race faces was not modified by participant race in the face regions.

**Fig. 10.**
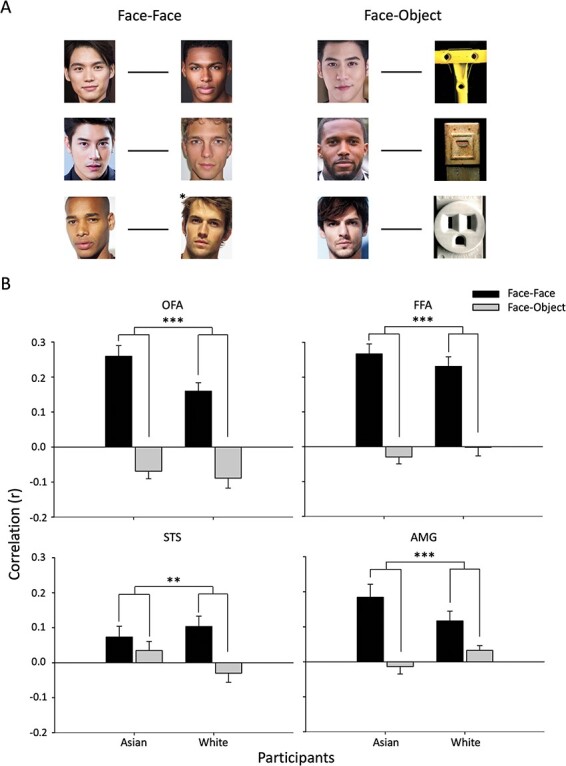
MVPA showing different spatial patterns of response to faces and pareidolic objects (A) The spatial pattern of response between different race faces (Face–Face) was compared to the spatial pattern between faces and pareidolic objects (Face–Object) in Asian and White participants. (B) The results reveal a significant effect of Category due to more similar patterns of response between faces (Face–Face) compared to the patterns between faces and objects (Face–Object). Error bars represent standard error of the mean. *** *P* < 0.001, ** *P* < 0.005.

Next, we analyzed the response to pareidolic objects. These objects have a face-like appearance, but they are not associated with a particular race. First, we measured the spatial pattern of response to pareidolic faces in the face regions (Figure 10). Our aim was to determine if they showed a similar or different pattern of response to faces. To do this, we compared the spatial pattern of response of faces from different races (Face–Face: Asian–Black, Asian–White, Black–White) with the spatial pattern of response between faces and pareidolic objects (Face–Object: Asian–Object, Black–Object, White–Object). The data were analyzed by a Category (Face–Face, Face–Object)*Participant (Asian, White) ANOVA. There was a significant effect of Category in the [OFA: *F*(1, 110) = 130.368, *P* < 0.001, η_G_^2^ = 0.542; FFA: *F*(1, 110) = 109.884, *P* < 0.001, η_G_^2^ = 0.500; STS: *F*(1, 110) = 9.251, *P* = 0.003, η_G_^2^ = 0.078; AMG: *F*(1, 110) = 28.896, *P* < 0.001, η_G_^2^ = 0.208) reflecting higher correlations for face–face than face–pareidolic object comparisons. There was no interaction between Category and Participant in the OFA [*F*(1, 110) = 2.334, *P* = 0.129, η_G_^2^ = 0.021], the FFA [*F*(1, 110) = 1.602, *P* = 0.208, η_G_^2^ = 0.014] and the STS [*F*(1, 110) = 2.820, *P* = 0.096, η_G_^2^ = 0.025] but there was a significant interaction in the AMG [*F*(1, 110) = 4.705, *P* = 0.032, η_G_^2^ = 0.041]. Overall, these findings show that the pattern of response to pareidolic objects is distinct from the pattern of response to faces in these face-selective regions, and this effect does not consistently vary between participant races.

**Fig. 11.**
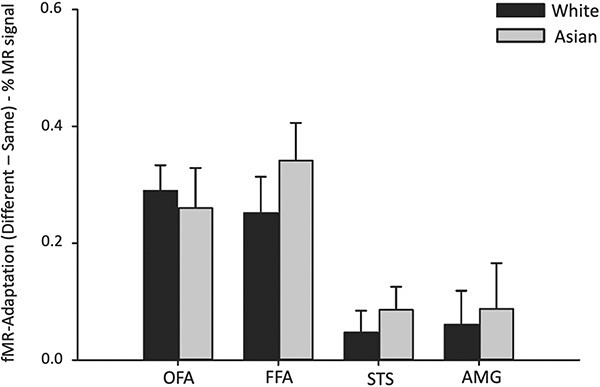
Similar neural responses to pareidolic objects in Asian and White participants. Adaptation to pareidolic objects was evident in the OFA and FFA. However, there was no difference in the magnitude of adaptation to pareidolic objects between White and Asian participants. Error bars represent standard error of the mean.

We also measured adaptation to pareidolic objects in the different face-selective regions. Figure 11 shows the adaptation to pareidolic objects in Asian and White participants. We found adaptation to pareidolic objects in the FFA [*F*(1, 55) = 3.008, *P* = 0.088, η_G_^2^ = 0.052] and OFA [*F*(1, 55) = 3.371, *P* = 0.072, η_G_^2^ = 0.058], but not in the STS [*F*(1, 55) = 1.457, *P* = 0.233, η_G_^2^ = 0.026] or AMG [*F*(1, 55) = 0.044, *P* = 0.836, η_G_^2^ = 0.001]. There was no interaction between Adaptation*Participant in any of the face regions [FFA: (1, 55) = 2.923, *P* = 0.093, η_G_^2^ = 0.050; OFA: *F*(1, 55) = 0.178, *P* = 0.675, η_G_^2^ = 0.003; STS: *F*(1, 55) = 1.681, *P* = 0.200, η_G_^2^ = 0.030; AMG: 0.201, *P* = 0.655, η_G_^2^ = 0.004]. Together, these analyses show that the face regions of Asian and White participants showed a similar level of adaptation to pareidolic objects.

## Discusssion

Our ability to categorize faces based on differences in race can play an important role in everyday social interactions (Tajfel *et al.*, 1971; Macrae and Bodenhausen, 2000; Meissner and Brigham, 2001). The aim of this study was to explore how differences in the faces of different races are represented in human and artificial neural networks.

First, we measured the similarity of face images from different races in an artificial neural network that has been trained in the recognition of faces (VGG-Face; Parkhi *et al.*, 2015). We found that the ability to discriminate faces from different races emerged in the later convolutional layers of the neural network and in the fully connected layers. This confirms the findings of a recent study that also used the VGG-Face (Tian *et al.*, 2021). These findings show that the structural differences that distinguish between faces from different races (Farkas *et al.*, 2005; Hill *et al.*, 1995) are evident in later stages of deep networks. The distinction between faces of different races in the top layers of the DCNN is perhaps not surprising in that race is an important cue to identity. Other studies have found that these layers of DCNNs also contain information about other attributes of the face, such as gender and viewpoint (O’Toole and Castillo, 2021). However, it is noteworthy that the transformation of a face from one race to another is not always detectable in the output layer of some DCNNs (Hancock et al., 2020). Interestingly, we also found that the ability of the DCNN to differentiate same-race faces from different-race faces was greater for White compared to Asian and Black faces, particularly in the later convolutional layers of the DCNN.

Next, we measured the ability of the DCNN to discriminate identity. As expected, the difference between same-identity faces and different-identity faces was greatest in the fully connected layers. The ability to discriminate identity from the DCNN in this study was similar to that for human observers (see Wang *et al.*, 2022). However, we found that the ability to discriminate identity was greater for White faces compared to Asian and Black faces. This bias for White faces is consistent with previous studies that have shown that face recognition algorithms have a bias toward faces that are used during training (Cavazos *et al*., 2020; Tian *et al.*, 2021). This also fits with developmental studies in which the bias toward the recognition of own-race faces increases with experience (Kelly *et al*., 2005; Chien *et al*., 2016) and by the fact that the ORE can be reversed or reduced if a person is exposed to another racial group during development (Sangrigoli *et al*., 2005; Sangrigoli and De Schonen, 2004). We also found that similarity between images was correlated with perceptual judgements of identity, particularly in the later convolutional and fully connected layers. Interestingly, we found that this correlation was greater for White faces compared to Asian or Black faces. Although DCNNs have a structure that is analogous to the human visual system (Krizhevsky *et al.*, 2012), the extent to which it operates in a similar way to the human visual system remains unclear (Kriegeskorte, 2015). The ability of the DCNN to predict human perceptual judgements and also show an own-race bias suggests a correspondence with the underlying representations in the human brain.

Next, we investigated whether there are different patterns of neural response to faces from different races in the human brain. Neuroimaging studies have identified a number of face selective regions (Haxby *et al.*, 2000): the OFA, FFA and pSTS region. The OFA is thought to be involved in the early perception of facial features and has a feed-forward projection to both the pSTS and the FFA (Pitcher *et al.*, 2007; Ishai, 2008; Davies-Thompson and Andrews, 2012). The connection between the OFA and pSTS is thought to be important in processing dynamic changes in the face, such as changes in expression and gaze, which are important for social interactions (Andrews and Ewbank, 2004; Engell and Haxby, 2007). The connection between the OFA and FFA is considered to be involved in the representation of invariant facial characteristics that are important for recognition (Rotshtein *et al.*, 2005; Weibert and Andrews, 2015). These regions interact with an extended network of regions in the brain that process faces, such as the AMG (Harris *et al.*, 2012).

Using MVPA, we compared the pattern of response to faces from the same race with the response to faces from different race. We used a LOPO MVPA approach in which we compared the pattern of response in one individual with the pattern from a group analysis of all other participants (Rice *et al.*, 2014; Watson *et al.*, 2014; Weibert *et al.*, 2018; Coggan *et al.*, 2019). This allowed us to ask how consistent the patterns of response were across different groups of participants. We were able to provide evidence that there were distinct patterns of response to faces from the same race in each of the face-selective regions. The largest effects of race were found at the early stages of processing in the OFA. These findings are consistent with the idea that OFA represents an earlier stage of processing in which the structural properties of the face are represented (Haxby *et al.*, 2000). However, it could be the case that this also might reflect that the pattern of response to faces is more consistent across participants in the OFA compared to other regions that could have a more idiosyncratic pattern of response. Nonetheless, these differences in representation may underlie our ability to categorize faces according to race.

Next, we asked whether own-race faces have a more similar pattern of response across participants when compared to other-race faces. We used a factorial analysis to ask whether the patterns of response across Asian participants were more similar to Asian faces compared to White faces and, conversely, were the patterns of response across White participants more similar for White faces compared to Asian faces. We found an own-race bias in the OFA and the FFA, but not in the STS or AMG. Previous MVPA studies have reported mixed findings on whether the spatial pattern of response in face regions can differentiate own-race and other-race faces (Ng *et al.*, 2006; Natu *et al.*, 2011). For example, Natu *et al.* (2011) showed that the pattern of response to own-race and other-race faces was evident, but only for a region of interest extended beyond the FFA. However, this study only measured responses from a relatively small number of participants, so it is possible that a significant difference may have become evident with a larger sample.

To further explore differences between own-race and other-race faces, we used an fMR-adaptation paradigm (Grill-Spector and Malach, 2001; Andrews and Ewbank, 2004). The prediction was that there should be greater adaptation to own-race faces. We found significant adaptation (reduced response to repetitions of identity) for Asian, White and Black faces in face-selective regions. The magnitude of the adaptation varied was generally higher in the OFA and FFA compared to the STS (see also Andrews and Ewbank, 2004), which presumably reflects the fact that the images did not vary in facial expression (see Harris *et al.*, 2012). However, we did not find that the magnitude of adaptation in any of the regions was modified by the race of the participant. These findings contrast with recent neuroimaging studies that found greater adaptation to own-race compared to other-race faces (Hughes *et al.*, 2019; Reggev *et al.*, 2020). A key difference between the current study and previous studies is our use of a factorial (cross-over) design in which both face race and participant race are varied simultaneously. This avoids the potential problem that results are due to differences in the stimulus set, rather than an other-race effect, per se (Natu and O’Toole, 2013). It is interesting to note that we did find adaptation was greater to Asian faces compared with White faces and Black faces. So, if our analysis had been restricted to Asian participants it would have shown levels of adaptation that would have been consistent with the behavioural other race effect. Another possible explanation for the lack of adaptation effects could be related to the time-scale of response to own-race and other-race faces. Natu *et al.* (2011) showed that there was an initial response advantage for own-race faces followed by greater adaptation of the own-race face response.

Together, we find some clear similarities in the response to faces from different races in human and artificial neural networks. Our results show that the ability to differentiate face race is evident in the fully connected layers of the DCNN and in the pattern of neural response across all face-selective regions. We find an own-race bias in the pattern of neural response of the OFA and FFA. That is, we find that Asian participants show more consistent patterns of response to Asian compared to White faces and White participants show more consistent patterns of response to White compared to Asian faces. This greater discrimination of own-race compared to other-race faces is similar to the pattern of results from the DCNN analysis in which there was a greater ability to discriminate race from White faces compared to Asian and Black faces, particularly in the later convolutional layers. This fits with the importance of the role of experience in the representation of faces in humans (Kelly *et al*., 2005; Chien *et al*., 2016; Sangrigoli *et al*., 2005; Sangrigoli and De Schonen, 2004) and DCNNs (Cavazos *et al*., 2020; Tian *et al.*, 2021). We also found that a DCNN was able to discriminate identity more efficiently for White faces compared to Asian and Black faces, consistent with behavioural studies in White participants (Malpass and Kravitz, 1969; Meissner and Brigham, 2001). However, an own-race bias did not extend to the univariate adaptation analysis of the neuroimaging results.

We also measured the response to objects that are perceived as faces (pareidolia). Although these objects give rise to the perception of a face, we did not expect that they would elicit a difference in response between the participants, as all participants would have a similar experience and perception of objects. Previous studies have found that pareidolic objects not only give rise to the perception of a face, but they also elicit face-like patterns of neural response (Taubert *et al.*, 2020; Wardle *et al.*, 2020). In a recent study, we found that the recognition of pareidolic objects was affected in developmental prosopagnosia, which again suggests similar underlying processes (Epihova *et al.*, 2022). Here, we found that there was significant adaptation to pareidolic objects in face-selective regions. However, we found that the spatial pattern of response to pareidolic objects was distinct from the pattern to faces. Together, these findings show that the neural response to pareidolic objects in face-selective regions shows some similarities, but also some differences, to the response to faces. Nevertheless, we did not find any effect of participant race on the MVPA or adaptation analysis of the pareidolic objects. This presumably reflects a similar exposure to objects across the different-race groups.

The focus of this study has been on the representation of face race. However, we are also able to categorize faces according to gender and age. Previous studies have shown that the ability to discriminate faces in these categories is dependent on structural differences in the faces (Burton *et al.*, 1993; Burt and Perrett, 1995). Accordingly, we would predict that this should be evident in the output of a deep neural network (see O’Toole and Castillo, 2021) or in the neural response of face regions in the human brain.

In conclusion, the results from this study show that structural differences in the faces from different races are found in the pattern of response of later layers of the deep neural networks and in face-selective regions. We also found that the pattern of response to own-race faces was more similar to other-race faces in the OFA and FFA. These results provide a neural correlate for both the ability to make categorical judgements about the race of faces and the behavioural advantage for recognizing own-race faces.
